# Hemodynamic and metabolic effects of estrogen plus progestin therapy in hypertensive postmenopausal women treated with an ACE-inhibitor or a diuretic

**DOI:** 10.1007/s00392-014-0755-6

**Published:** 2014-09-13

**Authors:** Anna Posadzy-Malaczynska, Katarzyna Rajpold, Lucyna Woznicka-Leskiewicz, Justyna Marcinkowska

**Affiliations:** 1 Department of Family Medicine, Poznan University of Medical Sciences, Przybyszewskiego 49, 60-355 Poznan, Poland; 2 Ist Department of Cardiology, Poznan University of Medical Sciences, Poznan, Poland; 3 Department of Hypertensiology, Angiology and Internal Diseases, Poznan University of Medical Sciences, Poznan, Poland; 4 Department of Computer Science and Statistics, Poznan University of Medical Sciences, Poznan, Poland

**Keywords:** Postmenopausal women, Hypertension, Estrogen plus progestin therapy, Pulse wave velocity, Renal plasma flow, Uric acid

## Abstract

**Objectives:**

The aim of the study was to assess the hemodynamic and metabolic actions of estrogen plus progestin therapy (EPT) in hypertensive, postmenopausal women treated with perindopril (ACEI) or hydrochlorothiazide (HCTZ). A group of normotensive postmenopausal women was also studied.

**Methods:**

100 hypertensive and 40 normotensive postmenopausal women were recruited for the study. The hypertensive females were randomly assigned to receive ACEI or HCTZ for 12 months. The patients of the ACEI group and the patients of the HCTZ group, as well as normotensives, were further subdivided into two subgroups each. One subgroup received estrogen plus progestin therapy (EPT+), the other subgroup received no hormone replacement (EPT−). Combined hormone replacement with transdermal patches releasing 17β-estradiol and norethisterone was used. Office and 24-hour ambulatory blood pressure was measured at baseline and during follow-up. Renal plasma flow (RPF) was measured using the clearance of [125I]-iodohippuran. Pulse wave velocity (PWV) was determined with an automatic device.

**Results:**

In normotensive postmenopausal women, transdermal estrogen plus progestin therapy increases RPF and insulin sensitivity, decreases PWV, decreases total and LDL cholesterol, and decreases uric acid serum levels. Perindopril (4 mg/day) and hydrochlorothiazide (25 mg/day) were equally effective in reducing blood pressure in postmenopausal, hypertensive subjects. In these females, perindopril increased RPF and decreased PWV and plasma insulin levels. These effects of the ACEI were not altered by estrogen plus progestin therapy. Hydrochlorothiazide decreased RPF and increased plasma insulin and uric acid concentrations in hypertensive subjects whom were not receiving estrogen plus progestin therapy.

**Conclusions:**

The unfavorable metabolic and hemodynamic actions of the diuretic were counteracted by estrogen plus progestin therapy. Concomitant estrogen plus progestin therapy may be a method to avoid unfavorable hemodynamic and metabolic effects of thiazide diuretics in hypertensive, postmenopausal women.

## Introduction

Despite the high prevalence of hypertension in middle-aged women, less than half of the patients receive adequate treatment, especially in older age groups when the risk of morbidity and mortality is the highest, due to coronary heart disease [1].

The reason for rising blood pressure levels at menopause is multifactorial and not completely understood. There are several hypotheses that include the role of sex hormones, the renin–angiotensin system, weight gain, and sympathetic activation [2].

Klungel et al. [3] observed an increased use of diuretics among women treated for high blood pressure than men (56.3 vs. 49.4 %). These results are similar to those published in the HOT study, in which more women than men received diuretic at the time of inclusion (36.4 vs. 29.8 %) [4]. The reason for the preferential use of diuretics in the treatment of hypertension in postmenopausal women is pathophysiological considerations, such as the increase of intravascular volume associated with sodium retention. The improvement of sodium sensitivity by thiazide diuretics causes the desired natriuretic effect. Additional benefit of their use is positive impact on calcium metabolism, which in the postmenopausal period is impaired. The Framingham study demonstrated much lower incidence of femoral fractures in women who were treated long-term with thiazide diuretics currently or in the past (>2 years) [5].

The use of thiazide diuretics, however, is associated with an increased serum uric acid. Therefore, as it is an independent factor of cardiovascular complications, this parameter and the unfavorable impact on lipid and carbohydrate metabolism may be considered as the cause of less benefits of treatment in a comparable age group of women and men.

Estrogen deficit in postmenopausal women seems to be the causative agent of severe vasoconstriction due to overexpression of the angiotensin AT_1_ receptor [6]. Plasma renin activity increases after menopause and the upregulation of angiotensin AT_2_ receptors and downregulation of angiotensin AT_1_ receptors at this time might influence response to therapy [7]. Therefore, for treatment of hypertension in postmenopausal women the most desirable medication would be such that could reverse or at least slow the disadvantages associated with vascular remodeling. The theory increased sensitivity of AT_1_ receptors to angiotensin II is considered to be one of the mechanisms of hypertension in postmenopausal women. Thus, the use of drugs that block the renin-angiotensin-aldosterone system fits perfectly in the pathogenesis of postmenopausal hypertension and the benefits of their use are presented in the clinical trials [8].

The newest statement on estrogen plus progestin therapy by the European and International Menopause societies reaffirms that this therapy is the most effective treatment for vasomotor symptoms and other symptoms of the menopause [9]. Overall, it is suggested that treatment with hormone therapy should be individualized. There is no standard approach to estrogen progestin therapy (EPT) decision-making. It depends on the severity of menopausal symptoms, impairment of quality of life, women’s personal risk-factor status, concomitant diseases, her personal preferences, and also before the age of 60 years and within 10 years after menopause when overall benefits are more likely to outweigh risks.

These points and pathophysiological aspects of hypertension after menopause captured an interest of the simultaneous influence of antihypertensive treatment and hormone substitution on hemodynamic and metabolic parameters. Renal blood flow is impaired in many postmenopausal women with hypertension [10]. It is conceivable that ACEI counteracts this impairment, whereas diuretics have no influence on it. The effects of EPT on hemodynamic and metabolic actions of ACEI and diuretics are unknown thus far. Therefore, a study was designed to assess the hemodynamic and metabolic actions of hormone replacement in hypertensive postmenopausal women treated with an ACEI or a diuretic. To assess the influence of EPT in subjects receiving no antihypertensive drugs, a group of normotensive postmenopausal women was also studied.

## Methods

Women with recently diagnosed, untreated essential hypertension in grade 1 or 2 according to ESH/ESC 2007 Guidelines [11] in the menopause were recruited to the study. They were qualified by a general practitioner or gynecologist as postmenopausal women aged 49–53, white. All women screened in the study reported full cessation of the menses for at least 12 months. The final menstrual period (FMP) is determined retrospectively. Among the surveyed women it was above 1 year. In determining this criterion, we used STRAW guidelines (stages of reproductive aging workshop) system [12]. The authors claim: twelve months of amenorrhea is considered to represent clinical menopause and is termed “postmenopause”. All the women had the expected postmenopausal rise of follicle-stimulating hormone levels (FSH 78.32 ± 8.73 IU/mL) and experienced flushes or other menopausal vasomotor symptoms. Therefore, according to the randomization, they were advised to undergo EPT and antihypertensive therapy. Women without hypertension but only with vasomotor symptoms were randomized for EPT or nothing. The exclusion criteria were: a first-degree relative having breast cancer, hyperplasia in endometrial biopsy, prior thromboembolic diseases, current or previous use of estrogen plus progestin therapy or contraceptives, diabetes, kidney failure, thyroid disease, and heart and other chronic diseases. All the screened women had a negative mammography in the 12 months prior to the study.

In the invitation letter, hypertensive women were asked to participate in a study on hypertension treatment and menopausal symptoms or menopausal symptoms only in women without hypertension. Participants gave their informed consent before the study. The study was conducted in accordance with The Declaration of Helsinki and was approved by the Ethics Committee of the University of Medical Sciences in Poznan. All women underwent a physical examination and biochemical screening at baseline.

The screening included 140 women with menopausal vasomotor symptoms, who met the inclusion but not exclusion criteria, and who had not yet been treated for hypertension. At two separate visits a week apart (wash-out period), hypertension was confirmed as the mean value of three office measurements ≥140/90 mmHg and <180/110 mmHg. Women with secondary hypertension or electrolyte disturbances (serum potassium levels) were excluded. Recruited women had no contraindications to transdermal estrogen plus progestin therapy or antihypertensive treatment. The control group consisted of 40 women with normal blood pressure (<140/90 mmHg two separate visits) measured in comparable conditions, who were also offered transdermal estrogen plus progestin therapy because of menopausal vasomotor symptoms.

Hypertensive women were then randomized at the beginning of the study (using sealed envelopes) to two therapeutic groups: with diuretic–hydrochlorothiazide 25 mg/day po (*n* = 50) or with ACEI– perindopril 4 mg/day po (*n* = 50) and to a group receiving estrogen plus progestin therapy (EPT+) and a group not treated with hormones (EPT−). Normotensive women were randomized only to EPT+ or EPT− groups. Estrogen plus progestin therapy consisted of transdermal patches releasing 17β-estradiol (0.05 mg/24 h) and norethisterone (0.25 mg/24 h) named Estracomb TTS^®^.

All participants had control measurements performed after 6 months and 1 year. At the end, 20 women did not complete the study (4 due to side effect of perindopril–cough, 6 needed additional hypotensive therapy, 2 on ACEI and 4 on HCTZ and 10 for no reason). All women completed the study in the normotensive group.

Finally, groups of 20 hypertensive women in each therapeutic option and 20 normotensive women, with and without EPT, were taken for statistical calculations in the order in which they have completed the 12-month follow-up period in the study.

### Measurements

Systolic (SBP) and diastolic (DBP) office blood pressure levels were measured after 5 min in sitting position, using a validated automatic device (Omron 705 CP) placed on the arm with the higher blood pressure level. Each time, the average of three readings was used. Ambulatory blood pressure measurements were performed in all patients at entrance into the study, as well as after 6 and 12 months using an automatic device (MOBIL-O-GRAPH). The devices were programmed to record the blood pressure during a 24 h period at the intervals of 30 min during daytime and intervals of 60 min at night.

Pulse wave velocity (PWV) was determined with an automatic device (Complior, Colson AS, Paris, France) by recording common carotid artery, as well as femoral artery pressure wave forms using a pressure-sensitive transducer (TY 306 Fukuda Denshi Co, Tokyo, Japan). Measurements were repeated during 20 cardiac cycles and the mean value was calculated. Details concerning the validation and the reproducibility of this method have been described [13]. In our laboratory, the variation coefficient was <6 %.

Renal plasma flow (RPF) was measured as the clearance of ^125^I-iodohippuran using a constant infusion technique with timed urine sampling [14, 15]. Plasma levels of glucose and insulin, as well as serum concentrations of total cholesterol and low-density cholesterol, were determined using fasting blood samples. Serum creatinine concentration was measured by use of the Jaffé reaction. Glomerular filtration rate (GFR) was estimated by the Cockroft-Gault equation for females.

### Statistical analysis

Student’s *t* test was used for comparisons continuous variables and normality of distribution of an analyzed feature in both populations. *t* test with the Cochran–Cox adjustment was used when variances of analyzed variables in both populations were different or Wilcoxon–Mann–Whitney test when there was no normal distribution.

Baseline characteristics were compared by use of ANOVA (for parametric variables) or the Kruskal–Wallis test (for non-parametric variables), respectively, followed by comparisons of the different groups in pairs. Means were compared using the Kruskal–Wallis test one-way analysis of ranks, followed by comparisons of groups of interest in pairs. If groups were significantly different, multiple comparison Dunn test was used.

Multiple linear regression models were calculated for selected parameters of the influence of HCTZ and EPT (hypertensive females). Also, models with interactions were calculated. If the regression coefficients β for the interactions of independent variables were significant, these models were used to interpret the variability of the dependent variable. *F* test verifies the statistical significance of all variables in the model. Similar multiple linear regression models were also calculated to indicate the hypertension and EPT influence.

Values represent mean ± standard deviation (SD) unless stated otherwise. A *p* value <0.05 was considered statistically significant.

## Results

Data at entry into the study show that the different subgroups of hypertensive patients were well matched (Table 1). The same is true for the two subgroups of normotensive women. As compared with the normotensive women, hypertensive women had higher office and ambulatory blood pressure levels, higher plasma insulin and uric acid levels a higher PWV, as well as a smaller nocturnal fall of systolic and diastolic blood pressure (Fall SBP_24_ Fall SBP_24_), lower GFR and RPF (Table 1). Lipid profile did not differ significantly between the subgroups. The lipid profile was also statistically equal between normotensive as well as hypertensive women. There were no differences at baseline in checked parameters between the ACEI and HCTZ groups (not shown in the Table).

**Table 1:** Baseline characteristics of the examined groups

	Hypertensives						Normotensives		
	ACEI			HCTZ			EPT+	EPT−	*p*
	EPT+	EPT−	*p*	EPT+	EPT−	*p*			
BP measurements									
mSBP (mmHg)	161.2 ± 9.6*	162.2 ± 9.8*	NS	159.7 ± 7.6*	160.4 ± 9.0*	NS	136.2 ± 2.5	136.5 ± 3.0	NS
mDBP (mmHg)	97.8 ± 2.4*	98.9 ± 3.1*	NS	97.1 ± 2.7*	98.4 ± 3.5*	NS	85.0 ± 2.6	85.6 ± 2.6	NS
mSBP_24_ (mmHg)	149.2 ± 6.3*	148.1 ± 6.9*	NS	149.0 ± 6.1*	148.5 ± 7.0*	NS	129.4 ± 2.7	129.9 ± 2.6	NS
mDBP_24_ (mmHg)	92.5 ± 1.5*	92.8 ± 1.9*	NS	92.7 ± 1.6*	92.9 ± 1.6*	NS	82.9 ± 2.3	83.6 ± 2.3	NS
Fall SBP_24_ (%)	7.0 ± 2.6*	7.1 ± 3.1*	NS	7.0 ± 2.2*	7.8 ± 3.2*	NS	9.9 ± 1.9	9.9 ± 1.9	NS
Fall DBP_24_ (%)	6.2 ± 1.8*	5.9 ± 1.8*	NS	6.4 ± 2.0*	6.5 ± 2.4*	NS	10.1 ± 2.1	10.6 ± 2.7	NS
Metabolic parameters									
Lipid profile									
Total cholesterol (mmol/L)	5.9 ± 0.4	6.0 ± 0.4	NS	5.8 ± 0.5	6.0 ± 0.4	NS	5.9 ± 0.5	5.8 ± 0.4	NS
LDL cholesterol (mmol/L)	3.7 ± 0.5	3.9 ± 0.4	NS	3.7 ± 0.5	3.9 ± 0.4	NS	3.8 ± 0.5	3.7 ± 0.4	NS
HDL cholesterol (mmol/L)	1.4 ± 0.1	1.4 ± 0.1	NS	1.4 ± 0.1	1.4 ± 0.1	NS	1.4 ± 0.1	1.3 ± 0.1	NS
Triglycerides (mmol/L)	1.7 ± 0.1	1.7 ± 0.1	NS	1.7 ± 0.1	1.7 ± 0.2	NS	1.6 ± 0.2	1.7 ± 0.1	NS
Glucose (mmol/L)	5.3 ± 0.6	5.5 ± 0.6	NS	5.2 ± 0.6	5.5 ± 0.6	NS	5.4 ± 0.6	5.3 ± 0.6	NS
Insulin (µI/L)	19.1 ± 6.4*	19.7 ± 7.3*	NS	19.6 ± 4.8*	20.3 ± 7.8*	NS	16.4 ± 5.1	17.0 ± 6.4	NS
Uric acid (mg/dL)	6.2 ± 2.1*	6.2 ± 2.0*	NS	6.2 ± 1.6*	6.4 ± 2.1*	NS	5.0 ± 1.4	5.0 ± 1.2	NS
Hemodynamic parameters									
PWV (m/s)	10.7 ± 1.7*	10.8 ± 1.4*	NS	10.8 ± 1.6*	10.8 ± 2.1*	NS	8.9 ± 1.5	8.6 ± 1.1	NS
RPF (mL/min)	421.9 ± 81.1*	423.7 ± 74.2*	NS	426.3 ± 66.0*	423.0 ± 75.1*	NS	488.7 ± 47.3	496.6 ± 45.0	NS
GFR (mL/min)	105.9 ± 7.5*	104.7 ± 7.7*	NS	106.9 ± 5.8*	105.2 ± 5.7*	NS	115.1 ± 5.2	114.8 ± 5.9	NS

*mSBP/mDBP* mean systolic/diastolic blood pressure in office measurement, *mSBP*_*24*_ */mDBP*_*24*_ mean systolic/diastolic blood pressure in 24-hour ambulatory monitoring, *Fall SBP*_*24*_ */Fall DBP*_*24*_ day-night fall of systolic/diastolic blood pressure in 24-hour ambulatory monitoring

* *p* < 0.05–0.0001 values that are, at baseline, significantly different in comparison to hypertensives ACEI (EPT+; EPT−) and HCTZ (EPT+; EPT−) vs. normotensives (EPT+; EPT), respectively

### The influence of estrogen plus progestin therapy on hemodynamic and metabolic measures in hypertensive postmenopausal women

Both perindopril and hydrochlorothiazide caused a similar decrease of blood pressure in hypertensive females (Table 2). After 6-(data not shown) and 12-month treatment, there were statistically significant differences between the EPT+ and EPT− subgroups in systolic and diastolic office blood pressure levels in ACEI treatment group. We did not observe such influence of EPT on ABPM (mSBP_24_ and mDBP_24_) among all treated women (Table 3). Multiple linear regression models have shown, after 12 months of hypotensive treatment, there were no significant differences in the nocturnal blood pressure fall (Fall SBP_24_ and Fall DBP_24_) by EPT treatment, despite its significant change in comparison to baseline values for ACEI and HCTZ subgroups. However, there was statistically significant lowering effect of the EPT on total and LDL cholesterol, as well as insulin levels in all hypertensive women (Table 4).

**Table 2:** The comparison of the change in systolic and diastolic blood pressure in ABPM between ACEI group and HCTZ group after 12 months of hypotensive treatment (both groups without EPT) showing no significant difference in lowering blood pressure by both drugs

	ΔSBP_24_ (mmHg)			ΔDBP_24_ (mmHg)		
	Mean	Day	Night	Mean	Day	Night
ACEI	21.1 ± 8.1	20.0 ± 7.8	25.0 ± 9.1	10.0 ± 2.7	7.0 ± 2.9	15.3 ± 6.0
HCTZ	21.9 ± 7.1	20.9 ± 7.5	26.4 ± 9.2	9.8 ± 2.6	7.9 ± 2.8	16.6 ± 3.8
*p* value	NS	NS	NS	NS	NS	NS

*ΔSBP*_*24*_ *;ΔDBP*_*24*_ difference in mean systolic; diastolic blood pressure in 24-hour ambulatory monitoring after 12 months in comparison to baseline

**Table 3:** The influence of EPT on mean values (±standard deviation) of different analyzed parameters after 12-month follow-up period

	Hypertensives						Normotensives		
	ACEI			HCTZ			EPT+	EPT−	*p*
	EPT+	EPT−	*p*	EPT+	EPT−	*p*			
BP measurements									
mSBP (mmHg)	132.2 ± 3.2*	134.3 ± 3.1*	<0.001	133.8 ± 5.6*	134.0 ± 4.0 *	NS	130.9 ± 3.4*	136.6 ± 2.2	<0.0001
mDBP (mmHg)	85.3 ± 2.3*	84.4 ± 2.6*	<0.05	85.3 ± 3.8*	85.1 ± 2.4*	NS	81.4 ± 2.4*	84.8 ± 2.2	<0.0001
ΔSBP_24_ (mmHg)	23.4 ± 5.8	21.1 ± 8.1	NS	22.1 ± 8.2	21.6 ± 7.1	NS	2.6 ± 2.7	2.2 ± 3.9	NS
ΔDBP_24_ (mmHg)	9.2 ± 2.5	10.0 ± 2.7	NS	8.7 ± 2.8	9.8 ± 2.6	NS	0.8 ± 2.9	0.5 ± 3.1	NS
mSBP_24_ (mmHg)	125.8 ± 2.1*	127.0 ± 3.3*	NS	126.9 ± 3.0*	126.7 ± 2.5*	NS	126.8 ± 2.8*	127.8 ± 3.4	NS
mDBP_24_ (mmHg)	83.8 ± 1.7*	82.8 ± 1.8*	NS	84.7 ± 2.0*	83.1 ± 1.8*	NS	82.1 ± 1.9	83.1 ± 1.8	NS
Fall SBP_24_ (%)	11.3 ± 1.7*	10.9 ± 2.5*	NS	9.6 ± 2.0*	8.6 ± 1.6	NS	11.4 ± 1.8*	9.5 ± 1.5	0.001
Fall DBP_24_ (%)	11.1 ± 1.6*	10.9 ± 1.8*	NS	9.5 ± 2.0*	9.0 ± 1.6	NS	11.8 ± 1.6	9.2 ± 1.8	0.001
Metabolic parameters									
Lipid profile (mmol/L)									
Total cholesterol	5.5 ± 0.3*	5.8 ± 0.3*	<0.01	5.8 ± 0.5	6.2 ± 0.8	NS	5.6 ± 0.2*	6.0 ± 0.5	<0.001
LDL cholesterol	3.4 ± 0.2*	3.6 ± 0.3*	<0.01	3.7 ± 0.5	4.1 ± 0.9	NS	3.4 ± 0.2*	3.9 ± 0.5	<0.01
HDL cholesterol	1.4 ± 0.1	1.4 ± 0.1	NS	1.4 ± 0.1	1.4 ± 0.1	NS	1.4 ± 0.1	1.4 ± 0.1	NS
Triglycerides	1.6 ± 0.1	1.7 ± 0.1	NS	1.7 ± 0.1	1.7 ± 0.1	NS	1.7 ± 0.2	1.7 ± 0.1	NS
Glucose (mmol/L)	5.4 ± 0.6	5.4 ± 0.6	NS	5.4 ± 0.6	5.6 ± 0.5	NS	5.4 ± 0.6	5.5 ± 0.6	NS
Insulin (µI/L)	14.4 ± 3.7*	16.8 ± 5.5*	NS	16.4 ± 5.1*	23.8 ± 8.4*	<0.01	12.7 ± 3.9*	16.6 ± 4.9	<0.01
Uric acid (mg/dL)	4.7 ± 1.3	5.3 ± 1.5	NS	5.4 ± 1.3	7.5 ± 2.3*	<0.001	3.8 ± 1.0*	5.1 ± 1.1	<0.001
Hemodynamic parameters									
PWV (m/s)	8.8 ± 1.6*	9.0 ± 1.5*	NS	9.3 ± 1.3*	11.5 ± 2.5	<0.01	7.8 ± 1.2*	8.8 ± 1.4	<0.05
RPF (mL/min)	519.4 ± 58.3*	488.7 ± 59.0*	NS	474.4 ± 57.5*	336.2 ± 49.1*	<0.001	538.8 ± 30.2*	485.0 ± 37.8	<0.0001
GFR (mL/min)	106.5 ± 7.1	106.8 ± 5.7	NS	105.2 ± 4.7	105.8 ± 4.9	NS	117.0 ± 5.0	114.5 ± 5.7	NS

*mSBP; mDBP* mean systolic; mean diastolic blood pressure in office measurement, Δ*SBP*_*24*_; Δ*DBP*_*24*_ difference in mean systolic; diastolic blood pressure in 24-hour ambulatory monitoring after 12 months in comparison to baseline, *mSBP*_*24*_ */mDBP*_*24*_ mean systolic/diastolic blood pressure in 24-hour ambulatory monitoring, *Fall SBP*_*24*_ */Fall DBP*_*24*_ day-night fall of systolic/diastolic blood pressure in 24-hour ambulatory monitoring

* *p* < 0.05–0.0001 values that are significantly different in comparison to baseline

**Table 4:** Multiple linear regression models of the influence of 12-month EPT and HCTZ (in comparison to ACEI) and their interaction in hypertensive women (*n* = 80)

	EPT	Hypotensive treatment (HCTZ)	HCTZ and EPT
Glucose	*β* = −0.11 (0.13)	*β* = 0.16 (0.13)	
	*p* = 0.37502	*p* = 0.21538	
mSBP	*β* = −0.50 (0.62)	*β* = 0.35 (0.62)	
	*p* = 0.42252	*p* = 0.57408	
mDBP	*β* = 1.28 (0.41)	*β* = 0.58 (0.41)	
	*p* = 0.00243*	*p* = 0.16132	
Fall SBP_24_	*β* = 0.69 (0.44)	*β* = −2.05 (0.44)	
	*p* = 0.1187	*p* = 0.00001*	
Fall DBP_24_	*β* = 0.30 (0.39)	*β* = −1.72 (0.39)	
	*p* = 0.44003	*p* = 0.00003*	
PWV	*β* = −0.25 (0.56)	*β* = 2.49 (0.56)	*β* = −1.97 (0.80)
	*p* = 0.656	*p* = 0.00003*	*p* = 0.01579*
GFR	*β* = −0.45 (1.26)	*β* = −1.20 (1.26)	
	*p* = 0.72251	*p* = 0.34487	
RPF	*β* = 30.70(17.74)	*β* = −152.55(17.74)	*β* = 107.50 (25.09)
	*p* = 0.08764	*p* = <0.000001*	*p* = 0.00005*
Insulin	*β* = −4.91 (1.35)	*β* = 4.51 (1.35)	
	*p* = 0.00048*	*p* = 0.00125*	
Total cholesterol	*β* = −0.32 (0.11)	*β* = 0.36 (0.11)	
	*p* = 0.00683*	*p* = 0.0022*	
LDL	*β* = −0.30 (0.12)	*β* = 0.38 (0.12)	
	p = 0.01194*	*p* = 0.00212*	
HDL	*β* = 0.004 (0.02)	*β* = −0.02 (0.02)	
	*p* = 0.85202	*p* = 0.28727	
Triglycerides	*β* = −0.11 (0.13)	*β* = 0.16 (0.13)	
	*p* = 0.37502	*p* = 0.21538	
Uric acid	*β* = −0.55 (0.52)	*β* = 2.21 (0.52)	*β* = −1.56 (0.73)
	*p* = 0.286821	*p* = 0.000051*	*p* = 0.03526*

*β* The regression coefficient and its error of estimation (SE_b_)

* Statistically significant value

During the study, the plasma glucose concentrations did not change in hypertensive patients treated with perindopril or hydrochlorothiazide (Table 3). Plasma insulin levels decreased significantly in the patients treated with perindopril, both in those subjects treated with and those without hormone replacement. At the end of the study, plasma insulin levels in the two subgroups treated with the ACEI (EPT+ vs. EPT−) did not differ significantly. In the hypertensive females treated with hydrochlorothiazide, plasma insulin level decreased significantly compared to baseline values only in the subgroup receiving estrogen plus progestin therapy to a level of 16.4 μU/mL at study end (Table 3). In contrast, plasma insulin and uric acid concentrations arose in those without estrogen plus progestin therapy (HCTZ; EPT− group) to 23.8 μU/mL and 7.5 mg/dL, respectively. The difference of plasma insulin and uric acid levels, between the two HCTZ subgroups (EPT+ vs. EPT−) was statistically significant (*p* < 0.01 and *p* < 0.001, respectively) at the end of the study.

At the end of the study, PWV of the perindopril group decreased significantly from baseline to similar levels both in patients with and in those without estrogen plus progestin therapy (Table 3). In the HCTZ group, PWV decreased significantly in the patients receiving estrogen plus progestin therapy. However, after 12-month treatment PWV was still higher in these patients than in females of the perindopril group receiving hormone replacement (9.3 vs. 8.8 m/s, *p* < 0.01—*p* value not shown in the table). In contrast to all other subgroups, PWV increased in patients without EPT during treatment with hydrochlorothiazide. At the end of the study, PWV was 11.5 m/s in these patients as compared with 9.3 m/s in patients of the HCTZ EPT+ group (*p* < 0.01) (Table 3).

Glomerular filtration rate (GFR) remained unchanged in all subgroups during the study (Table 3). RPF increased significantly compared to baseline values in the patients treated with perindopril, both in those with and those without estrogen plus progestin therapy (Table 3). At the end of the study, RPF in the two subgroups did not differ between ACEI (EPT+ vs. EPT−) groups. RPF also increased in the patients treated with hydrochlorothiazide and received EPT. However, RPF was substantially decreased in the patients of the hydrochlorothiazide subgroup without EPT. At the end of the study, RPF in the HCTZ group was 474.4 mL/min in EPT+ comparing with 336.2 mL/min in EPT− (*p* < 0.001). In both hypertensive subgroups treated with EPT, the changes of PWV and of RPF were inversely correlated (*r* = −0.67, *p* < 0.01 in the patients of the perindopril group treated with EPT; *r* = −0.43, *p* < 0.05 in the patients of the HCTZ group who received EPT).

### The effects of estrogen plus progestin therapy on hemodynamic and metabolic measures in normotensive postmenopausal women

In comparison to baseline values, office systolic and diastolic blood pressure levels decreased in the normotensive females treated with hormone replacement. The same was concluded for the systolic ambulatory blood pressure. EPT+ and EPT− groups differed significantly in office BP as well as nocturnal fall of BP (respectively, *p* < 0.0001 and 0.001). Such a change was not observed in ABPM (mSBP_24_ and mDBP_24_). Moreover the influence of EPT on BP changes (∆SBP_24_ and ∆DBP_24_) turned out to be insignificant (Table 3).

At the end of the study, there were statistically significant differences between the EPT+ and EPT− subgroups in total and LDL cholesterol levels (*p* < 0.001 and *p* < 0.01, respectively). These values were significantly changed from baseline only in EPT subgroups.

Plasma glucose levels remained unchanged during the study in normotensive women with EPT as well as in women without EPT (Table 3). However, plasma insulin and uric acid concentrations decreased in the estrogen plus progestin therapy, whereas these measurements remained unaltered in the subgroup without EPT. At the end of the study, plasma insulin concentration was 12.7 μU/mL in the EPT+ subgroup as compared with 16.6 μU/mL in the EPT− subgroup (*p* < 0.01). Uric acid levels were also significantly lower by EPT (*p* < 0,001).

During the study, PWV decreased in normotensive females given estrogen plus progestin therapy, whereas PWV remained unaltered in women without EPT (Table 3). After 12 months, PWV was 7.8 m/s in EPT+ and 8.8 m/s in EPT−. The difference was statistically significant (*p* < 0.05). GFR remained unchanged during the study in both subgroups (Table 3). In contrast, RPF increased in EPT+ and remained unchanged in EPT− in comparison to baseline values. At the end of the study, RPF was 538.8 mL/min in EPT+ and 485 mL/min in EPT− (*p* < 0.0001).

**Fig. 1:**
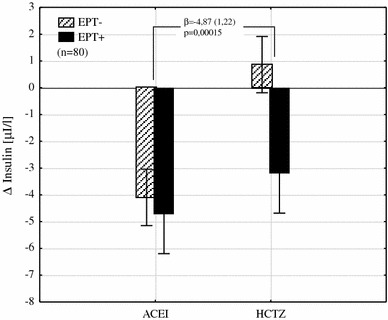
The interaction between the type of hypotensive treatment and EPT on insulin change (*unfilled triangles*) shows its reduction by an average of 4.87 µI/L in women with EPT added to HCTZ (*β* the regression coefficient and its error of estimation, *EPT* estrogen plus progestin therapy, *ACEI* angiotensin converting enzyme inhibitor, *HCTZ* hydrochlorothiazide)

**Fig. 2:**
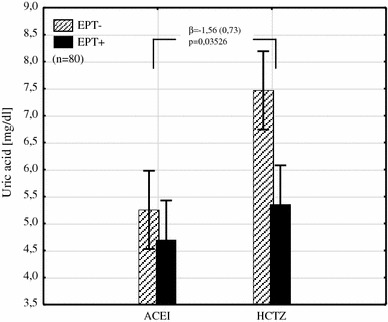
The interaction between the type of hypotensive treatment and EPT on uric acid plasma level shows its reduction by an average of 1.56 mg/dL in patients with EPT added to HCTZ (*β* the regression coefficient and its error of estimation, *EPT* estrogen plus progestin therapy, *ACEI* angiotensin converting enzyme inhibitor, *HCTZ* hydrochlorothiazide)

**Fig. 3:**
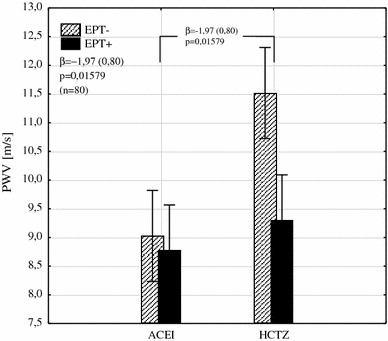
The interaction between the type of hypotensive treatment and EPT on PWV shows its reduction by an average of 1.97 m/s in patients with EPT added to HCTZ (*β* the regression coefficient and its error of estimation, *PWV* pulse wave velocity, *EPT* estrogen plus progestin therapy, *ACEI* angiotensin converting enzyme inhibitor, *HCTZ* hydrochlorothiazide)

**Fig. 4:**
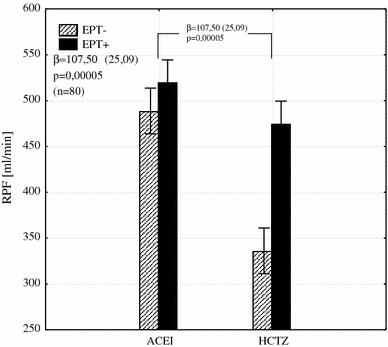
The interaction between the type of hypotensive treatment and EPT on RPF shows its increase by an average of 107.5 mL/min in patients with EPT added to HCTZ (*β* the regression coefficient and its error of estimation, *RPF* renal plasma flow, *EPT* estrogen plus progestin therapy, *ACEI* angiotensin converting enzyme inhibitor, *HCTZ* hydrochlorothiazide

**Fig. 5:**
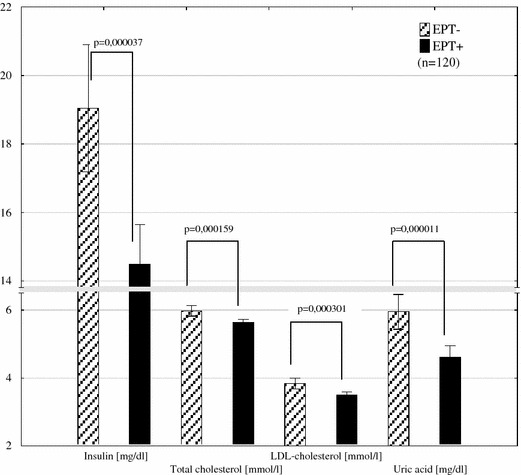
The comparison of plasma levels of insulin, total cholesterol, LDL cholesterol, uric acid between women treated with (*n* = 60) or without EPT (*n* = 60) (*EPT* estrogen plus progestin therapy)

In multivariate analysis, we revealed significant interactions between estrogen plus progestin therapy and the type of hypotensive treatment in metabolic and hemodynamic parameters such as insulin changes (insulin delta is not shown in the table) (Fig. 1) and uric acid plasma concentrations (Fig. 2; Table 4) and as well as PWV and RPF (Table 4; Figs. 3, 4). Moreover levels of insulin, total cholesterol, LDL cholesterol, and uric acid were significantly lower in all women treated with EPT (Table 5; Fig. 5).

**Table 5:** Multiple linear regression models of the influence of hypertension and 12-month EPT on metabolic profile in all examined women (*n* = 120)

	Hypertension	EPT
Glucose	*β* = −0.03 (−0.02)	*β* = −0.09 (−0.08)
	*p* = 0.796689	*p* = 0.375755
Insulin	*β* = 3.18 (0.24)	*β* = −4.55 (−0.36)
	*p* = 0.005481*	*p* = 0.000037*
Total cholesterol	*β* = 0.03 (0.02)	*β* = −0.34 (−0.34)
	*p* = 0.7821	*p* = 0.000159*
LDL	*β* = 0.04 (0.03)	*β* = −0.34 (−0.32)
	*p* = 0.687098	*p* = 0.000301*
HDL	*β* = −0.01 (−0.06)	*β* = 0.004 (0.02)
	*p* = 0.540434	*p* = 0.789504
Triglycerides	*β* = −0.003 (−0.01)	*β* = −0.01 (−0.05)
	*p* = 0.903618	*p* = 0.564273
Uric acid	*β* = 1.23 (0.31)	*β* = −1.34 (0.29)
	*p* = 0.000115*	*p* = 0.000011*

*β* the regression coefficient and its error of estimation (SE_b_)

* Statistically significant value

## Discussion

Menopause is associated with enhanced progression of atherosclerosis [16]. The stiffness of the female aortic wall increases more rapidly during the menopausal years than the changes at the same time of life in men [17].

Our study addresses the EPT timing hypothesis that the beneficial vascular effects of EPT after menopause will only accrue if such therapy is given before advanced atherosclerosis develops [18–20].

In the WHI study, the average age of randomized women was 67 years, whereas our population was much younger and thus free of the complications of menopause. We believe that this is the best time (shortly after menopausal transition) to demonstrate the impact of early estrogen progestin therapy and appropriate antihypertensive treatment to reverse negative aspects of menopause on the cardiovascular and metabolic status [21, 22].

The study shows that estrogen plus progestin therapy has several favorable effects in normotensive postmenopausal women. Office blood pressure fell slightly, nocturnal fall of SBP increased significantly reaching the standard values (>10 %), and PWV decreased, while RPF increased. The fall of PWV is partially caused by the decrease of blood pressure and restoring the nocturnal fall of BP, but also because of other reasons. The concomitant increase of RPF, however, suggests that structural changes in the vessel walls may contribute. This assumption is supported by the findings of Hayward et al. [23].

The improvement of elastic properties of the aorta may result from increase of vasa vasorum flow which contribute substantially to the nutrition of the outer layers of the thoracic aorta and therefore modulate the structure of the aortic wall [24, 25].

During EPT in normotensives plasma insulin levels were decreased, while plasma glucose concentrations remained unchanged, indicating enhanced insulin sensitivity. Renal parameters together with metabolic profile improvement (decrease of total cholesterol, its LDL fraction and uric acid) were caused by beneficial influence of EPT introduced in women early after menopausal transition (aged 49–53 years).

With the doses used in our study, the perindopril and the hydrochlorothiazide had similar antihypertensive effects on blood pressure in ABPM in hypertensive women. Therefore, we suspect that a complex mechanism played a role in improving hemodynamic parameters after application of transdermal EPT.

Performing blood pressure measurements (office and ABPM) allowed us to demonstrate the usefulness of ABPM in effect on circadian profile and also revealed that differences in the office measurements turned out to be insignificant in ABPM. In contrast, improvement in nocturnal blood pressure fall in the “net” dimension after application of EPT and antihypertensive treatment becomes favorable in the aspect of reduction of cardiovascular events.

Among women with hypertension, nocturnal pressure fall of systolic and diastolic blood pressure increased significantly and was restored (>10 %) in the group treated with ACEI. Diuretic treatment did not restore the nocturnal pressure fall, but the addition of hormone substitution increased significantly both systolic and diastolic falls. The absence of nocturnal blood pressure fall is recognized in the literature as a factor correlating with increased risk of cardiovascular complications [26–28]. In postmenopausal women the percentage of “non-dippers” is higher, at around 40 % compared with men [29]. Some authors have expressed the view that women “non-dippers” are more likely to develop cardiovascular complications of hypertension than men [30, 31]. Lack of improvement of the night fall in the group treated with HCTZ may be due to unfavorable effects of the diuretic itself on the pressure profile, which can only be assessed by monitoring the pressure all day.

Messerli et al. [32] found in their meta-analysis that HCTZ lowers BP well during daytime when patients are seen in the physician’s office, but has less effect during the night and early morning hours. So it appeared useful to make ABPM measurements that documented the daily pressure profile improvement on EPT in women treated with HCTZ. This is because this diuretic is still often used as a first-line drug in the treatment of hypertension in postmenopausal women, not only because of the pathomechanism of hypertension in this period (the retention of sodium in the body), but also because of the desired beneficial effect on the calcium metabolism.

Angiotensin converting enzyme inhibitor, in addition to its antihypertensive action had several other favorable effects in hypertensive postmenopausal females [33]. The drug decreased PWV, not only because of the fall of blood pressure but also because of other than hypotensive effects. RPF was increased and plasma insulin levels were decreased in the patients without hormone replacement during treatment with the ACEI. Estrogen plus progestin therapy did not significantly alter PWV, RPF, and plasma insulin concentrations in these women. In contrast to the ACEI, HCTZ had some unfavorable effects in hypertensive postmenopausal women who received no estrogen plus progestin therapy. PWV did not change in spite of a significant fall of blood pressure. RPF was decreased and plasma insulin levels rose. Unfavorable influence of factors on the elastic properties of vessel walls and their remodeling changes with age and female hormone loss during menopausal transition, and it may predominate on hypotensive effect of HCTZ on hemodynamic parameters which finally causes their deterioration. Estrogen plus progestin therapy counteracts these effects.

There are conflicting reports concerning the influence of estrogens on plasma insulin levels in normotensive females [34, 35]. In our study, estrogen plus progestin therapy decreased plasma insulin concentrations in normotensive postmenopausal women and antagonised the thiazide-induced rise of plasma insulin in hypertensive females. High insulin levels cause proliferation of vascular smooth muscle cells [36, 37] and increase calcium influx into these cells [38] resulting in increased vascular media thickness and vascular stiffness [39]. This could be the cause of unchanged PWV in spite of decreased blood pressure values as well as of the decrease of RPF in our patients treated with HCTZ and receiving no estrogen plus progestin therapy.

In a meta-analysis including 103,268 men and 87,349 women, antihypertensive therapy provided similar protection against cardiovascular events in both sexes. Furthermore, in this meta-analysis, there was no evidence that ACEI was superior to diuretics in preventing cardiovascular complications in hypertensive females [40]. One should, however, keep in mind that for the trials included in the meta-analysis both pre- and postmenopausal women were recruited. In our study with postmenopausal women receiving no estrogen plus progestin therapy, the ACEI perindopril—in addition to its antihypertensive action—decreased plasma insulin concentrations and increased RPF. The diuretic HCTZ had the opposite effects. The unfavorable actions of HCTZ on plasma insulin levels and RPF were counteracted by estrogen plus progestin therapy. In contrast, EPT did not significantly influence RPF and plasma insulin concentrations in patients treated with perindopril. The multivariate analysis showed that the positive cumulative effect of EPT and HCTZ seems to be an attractive concept for the abolition of both the adverse effects of the thiazide diuretic on metabolic and hemodynamic parameters, and concomitant treatment of menopausal symptoms. The benefits may even surpass antihypertensive therapy with ACE inhibitors and EPT. This transdermal combination in the present study has been found to have positive effects on lipid metabolism. The difference in total and LDL cholesterol levels between groups (lower levels in EPT group with ACEI and in normotensives and not changed in HCTZ group) after 12 months may partially explain the beneficial effects of estrogen plus progestin therapy on arterial compliance and stay in line with impact on cardiovascular endpoints.

Transdermal application of low doses of 17β-estradiol and norethisterone was used as estrogen plus progestin therapy in our study. This provides sustained release and more constant blood levels of the drugs than those that could be obtained with oral application. Furthermore, the transdermal application avoids a first pass effect and, therefore, interactions with agents affecting drug metabolising enzymes in the liver [41].

## Conclusions

In conclusion, our study shows that transdermal estrogen plus progestin therapy increases RPF and insulin sensitivity as well as decreases PWV, total and LDL cholesterol, uric acid serum levels in normotensive postmenopausal women. Perindopril (4 mg/day) and hydrochlorothiazide (25 mg/day) were equally effective in reducing blood pressure in hypertensive postmenopausal females. In these patients, perindopril increased RPF and decreased PWV and plasma insulin levels. These effects of the ACEI were not altered by estrogen plus progestin therapy. HCTZ decreased RPF and increased plasma insulin and uric acid concentrations in hypertensive, postmenopausal women not receiving estrogen plus progestin therapy. These unfavorable actions of the diuretic were counteracted by estrogen plus progestin therapy. Concomitant estrogen plus progestin therapy may be a method to avoid unfavorable hemodynamic and metabolic effects of thiazide diuretics in hypertensive postmenopausal women.

## Abbreviations

ACEI: Angiotensin converting enzyme inhibitor
BMI: Body mass index
BP: Blood pressure
EPT: Estrogen plus progestin therapy
FMP: Final menstrual period
GFR: Glomerular filtration rate
HCTZ: Hydrochlorothiazide
mSBP: Mean systolic blood pressure office measurement
mDBP: Mean diastolic blood pressure office measurement
mSBP_24_: Mean systolic blood pressure in 24-hour ambulatory monitoring
mDBP_24_: Mean diastolic blood pressure in 24-hour ambulatory monitoring
∆SBP_24_: Difference in mean systolic blood pressure in 24-hour ambulatory monitoring after 12 months in comparison to baseline
∆DBP_24_: Difference in mean diastolic blood pressure in 24-hour ambulatory monitoring after 12 months in comparison to baseline
PWV: Pulse wave velocity
RPF: Renal plasma flow
STRAW: Stages of reproductive aging workshop
